# A Novel Circular RNA CircRFX3 Serves as a Sponge for MicroRNA-587 in Promoting Glioblastoma Progression via Regulating PDIA3

**DOI:** 10.3389/fcell.2021.757260

**Published:** 2021-12-07

**Authors:** Tong Li, Jianguo Xu, Yi Liu

**Affiliations:** The Department of Neurosurgery of West China Hospital of Sichuan University, Chengdu, China

**Keywords:** circRFX3, miR-587, PDIA3, pathway, molecular target

## Abstract

An increasing number of studies have indicated that circular RNAs (circRNAs) participate in the progression of numerous tumors. However, the functions of circRNAs in glioblastoma (GBM) remain largely unknown. In this study, we focused on a novel circRNA (hsa_circRFX3_003) that was spliced from RFX3, which we named circRFX3. We confirmed that the expression of circRFX3 was substantially increased in GBM cell lines and clinical GBM tissues. The results of a series of overexpression and knockdown assays indicated that circRFX3 could boost the proliferation, invasion, and migration of GBM cells. By performing dual-luciferase reporter gene and RNA pull-down assays, we verified that circRFX3 could sponge microRNA-587 (miR-587) to exercise its function as a competing endogenous RNA (ceRNA) in the development of GBM. In addition, PDIA3 was proven to be a downstream target of miR-587 and to regulate the Wnt/β-catenin pathway. In conclusion, circRFX3 could act as a cancer-promoting circRNA to boost the development of GBM and regulate the miR-587/PDIA3/β-catenin axis. This study might provide a novel target for the treatment of GBM with molecular therapy.

## Introduction

In the central nervous system, gliomas are the most common tumors, accounting for approximately 80% of intracranial tumors (Alexander and Cloughesy, 2017). Glioblastomas (GBMs), which account for half of gliomas, are the most malignant (Janjua et al., 2021). Even when treated aggressively, the median survival time of GBM patients is only 14–16 months (Reardon et al., 2006). To date, molecular therapies have not shown satisfactory treatment outcomes for GBM in clinical practice. Hence, it is important to identify novel effective targets in the development of GBM (Goodall and Wickramasinghe, 2021).

Circular RNA (circRNA) is a noncoding RNA (ncRNA) that is diffusely expressed in cells. circRNA is produced in the progression of back-splicing and has a covalently closed loop structure, which is the basis of its stable existence (Kristensen et al., 2019). As a number of studies have indicated, circRNAs can function as competing endogenous RNAs (ceRNAs) that sponge miRNAs or bind to proteins to regulate the biological behavior of cells (Thomson and Dinger, 2016). There is convincing evidence verifying that circRNA plays an essential role in tumor progression—for example, circHMGA2 can regulate the development of lung cancer cells through the miR-1236-3p/ZEB1 axis (Yu et al., 2021a). Hsa_circ_0001666 can boost the development of thyroid carcinoma upregulation of ETV4 (Qi et al., 2021), and circHIPK3 can influence myoblast progression by regulating the miR-7/TCF12 axis (Gao et al., 2021). In addition, circRNAs also participate in the progression of GBM, which has drawn the attention of many scholars—for instance, circPITX1 promotes the progression of GBM by regulating the miR-584-5p/KPNB1 axis (Cao et al., 2021).

In the present study, with the help of bioinformatics, we found a novel circRNA (circRFX3, hsa_circRFX3_003) that has never been studied before and verified it as overexpressed in GBM cell lines and clinical tumor tissues. Furthermore, the expression of circRFX3 was negatively correlated with the overall survival time of GBM patients. As demonstrated by experimental outcomes, circRFX3 was located mainly in the cytoplasm, and the results of a series of overexpression and knockdown assays showed that circRFX3 regulated the proliferation, invasion, and migration of GBM cells *in vitro* and *in vivo*. Subsequently, dual-luciferase reporter gene and RNA pull-down assays were carried out to confirm that miR-587 binds directly to the binding sites of circRFX3 and PDIA3 mRNA. Additionally, we also demonstrated that PDIA3 could substantially influence and regulate the malignant biological behavior of GBM and regulate the Wnt/β-catenin pathway. Ultimately, the circRFX3/miR-587/PDIA3/β-catenin pathway was confirmed. The results of our investigation could provide a new target for molecular therapy and help improve clinical strategies.

## Methods and Materials

### Cell Lines and Clinical Tumor Samples

U118, U251, U87, and A172 cell lines were purchased from the American Type Culture Collection (Rockville, MD, United States). The cell lines were cultured in 1640 medium (Servicebio, Wuhan, China) containing 10% fetal bovine serum (Servicebio, Wuhan, China), streptomycin (100 mg/ml), and penicillin (100 U/ml). The human astrocyte (HA) cell line was purchased from ScienCell (San Diego, CA, United States). Primary human astrocytes (PHA1 and PHA2) were purified from the non-functional brain tissue collected from two patients with severe brain trauma.

The collection of clinical samples was approved by the Ethics Committee of West China Hospital, and written permission was acquired from all the patients involved in this research. Forty-two paired GBM samples were utilized to verify the microarray analysis results. All tissues were obtained from West China Hospital between February 2016 and November 2019. Each tumor sample was histologically identified and diagnosed as glioblastoma multiforme.

### Bioinformatic Analysis

The Cancer Genome Atlas (TCGA) databases (http://cancergenome.nih.gov/) were used to obtain patient statistical data and clinical information. Volcano plot filtering was used to identify differentially expressed circRNAs that were statistically significant between the two groups. Cluster analysis was performed with the heatmap R package (https://cran.r-project.org/web/packages/pheatmap/index.html). The batch effect was removed with the Sva R package. We analyzed the connections between genes and corresponding overall survival by drawing Kaplan–Meier survival curves in the survival package. We performed statistical analysis and drew graphics with the R software (R version 3.3.2). The obtained array images were analyzed with the Agilent Feature Extraction software (version 11.0.1.1).

### RNA Extraction and Quantitative Real-Time PCR

For the extraction of total RNA from GBM cells and clinical tissues, we used TRIzol (Thermo Fisher Scientific, Waltham, MA, USA) following the protocols of the manufacturer. We used SuperScript™ III Platinum™ SYBR™ Green (Thermo Fisher Scientific, Waltham, MA, United States) to generate the cDNA of mRNA and circRNA. We carried out quantitative real-time PCR (qRT-PCR) with SuperScript™ III Platinum™ SYBR™ Green (Thermo Fisher Scientific, Waltham MA, United States). GAPDH and U6 were used as internal controls. The TaqMan™ MicroRNA Reverse Transcription Kit (Thermo Fisher Scientific, Waltham, MA, United States) was utilized to obtain the cDNA of microRNA. The TaqMan™ MicroRNA Assay (Thermo Fisher Scientific, MA, United States) was used to carry out qRT-PCR. qRT-PCR was carried out utilizing ABI 7500 real-time PCR systems (Applied Biosystems, Foster City, CA, United States), and we calculated the RNA relative expression levels by using the 2-ΔΔCt method. The primers were obtained from Sangon Biotech (Shanghai, China). microRNA reverse primers were obtained from the TaqMan™ MicroRNA Assay (Thermo Fisher Scientific, Waltham, MA, United States). The primer sequences used for PCR amplification were as follows:

**Table:** 

circRFX3	Forward primer	5′-TGT​AAA​TGA​TGG​GGG​TGG​AGA​A-3′
	Reverse primer	5′-TTT​TCC​ATA​GCA​TTG​ACA​ACC​AT-3′
RFX3	Forward primer	5′-CAC​AGG​CTC​GAC​AGT​GAC​C-3′
	Reverse primer	5′-GCA​CAG​TCT​GTA​CCT​GCT​GTA-3′
PDIA3	Forward primer	5′-GCC​TCC​GAC​GTG​CTA​GAA​C-3′
	Reverse primer	5′-GCG​AAG​AAC​TCG​ACG​AGC​AT-3′

### shRNAs, Plasmids, and Establishment of Stabilized Cell Lines

We amplified the circRFX3 sequence and inserted it into the circRNA overexpression vector pLCDH-ciR (Geneseed, Guangzhou, China). This insertion was confirmed by sequencing and through restriction enzyme sites BamHI and EcoRI. We obtained three circRFX3 short hairpin RNAs (shRNAs) from Geneseed (Guangzhou, China). We incubated U87 cells with circRFX3 overexpression lentivirus for 48 h and selected stable cell lines by using puromycin (5 μg/ml) for 28 days. The circRFX3 shRNA sequences were as follows:

**Table:** 

sh-1	5′-GTG​GAG​AAG​ATA​AAT​GAG​TAT-3′
sh-2	5′-GGG​GGT​GGA​GAA​GAT​AAA​TGA-3′
sh-3	5′-GGG​GTG​GAG​AAG​ATA​AAT​GAG-3′

### Actinomycin D and RNase R Treatment

Actinomycin D (2 mg/ml) was added to the culture medium to block transcription, and we used dimethylsulfoxide (Sigma-Aldrich, St. Louis, MO, United States) as a negative control. For RNase R treatment, we incubated total RNA at 37°C for 30 min with or without RNase R (4 U/mg) (YEASEN, Shanghai, China). We performed qRT-PCR to detect the expression levels of circRFX3 and RFX3 mRNA after treatment with actinomycin D and RNase R.

### Fluorescence *In Situ* Hybridization

The circRFX3 probe was labeled by Cy3. After culturing cells in medium with 10% FBS for 24 h, we used 4% paraformaldehyde to fix the cells at 23°C for 20 min. After that, we washed the cells with PBS. Then, we utilized 0.5% Triton-X-100 to incubate the cells at 23°C for 5 min and used a prehybridization buffer (GenePharma, Suzhou, China) to incubate the cells at 37°C for 30 min. Furthermore, we used a hybridization buffer (GenePharma, Suzhou, China) with probes to culture the cells overnight at 37°C. After that, we used 4× saline-sodium citrate (SSC), 2× SSC, and 1× SSC to wash the cells at 42°C for 3 × 5 min. We used DAPI to stain the nuclei at 23°C for 10 min. The sequence of the probe of circRFX3 was 5′-CCC​CAC​CTC​TTC​TAT​TTA​CTC​ATA-3′.

### Isolation of RNAs From Nuclear and Cytoplasmic Fractions

Following the protocols of the manufacturer, we used a Cytoplasmic & Nuclear RNA Purification Kit (Invitrogen, Carlsbad, CA, United States) to isolate the nuclear and cytoplasmic fractions. The cells were harvested and then lysed with a cell fractionation buffer. Next, we separated nuclear and cytoplasmic fractions through centrifugation. The cytoplasmic fractions in the supernatant were collected and transferred into fresh RNase-free tubes. We used a cell disruption buffer to lyse the nuclear pellet. We mixed 2× lysis/binding solution with the cytoplasmic fractions and nuclear lysate and added 100% ethanol. The filter cartridge was used to absorb the mixture. We used an elution solution to elute nuclear and cytoplasmic RNAs. GAPDH and U6 small nuclear RNA (snRNA) were used, respectively, as controls for the cytoplasmic and nuclear RNAs.

### CCK-8 Assay

We harvested and resuspended the logarithmic phase cells and spread 1,000 cells in each well of a 96-well plate. After 24 h, the cells successfully adhered to the bottom of the wells, and we added CCK-8 (Abcam, Cambridge, United Kingdom) to the samples. The blank control was a well containing only the medium and CCK-8 solution. The absorbance values of the wells at 450 nm were measured at intervals of 24 h for 4 days.

### EdU Assay

We seeded and cultured the cells in a 96-well plate. Then, we used EdU (Beyotime, Shanghai, China) to treat the cells. After incubating for 2 h at 37°C, the cells were fixed through 4% paraformaldehyde for 30 min, and we utilized 0.5% Triton-X-100 to incubate the cells for 20 min. Hoechst was used to stain the nuclei. We calculated the proliferation rate based on the protocols of the manufacturer. Images of three randomly selected areas of each group were taken with a fluorescence microscope.

### Colony-Formation Assay

We suspended the cells and seeded them into the wells of 6-well plates at 1 × 10^3^ cells per well. After incubating at 37°C with 5% CO_2_ for 14 days, the colonies were fixed through 4% paraformaldehyde for 30 min, and we utilized crystal violet to stain the colonies for 30 min. Then, we recorded the number of colonies in each well.

### Transwell Invasion and Cell Wound-Healing Assays

We performed transwell experiments with Transwell chambers (Millipore, Billerica, MA, United States). In invasion experiments, we collected modified GBM cells and utilized serum-free medium to resuspend them. The density was 8 × 104/ml. Next, 200-μl cell suspension was dripped into the upper part of the chamber. Then, we added 700 μl medium containing 10% FBS to the lower compartment, which was then incubated for 24 h. We removed the chamber and gently wiped the cells on the upper chamber. Then, the cells on the other side of the membrane were fixed with 4% paraformaldehyde solution, stained for 30 min with crystal violet and gently washed three times with PBS. After the membrane dried, we used a microscope to observe and record the cells and randomly selected six fields of view for recording. The mean number of cells stained was considered the value of invading cells.

For the cell wound-healing assay, we incubated the cells in six-well plates to full confluence. Then, we used a 200-μl micropipette tip to scratch the middle of the wells. After that, we used serum-free medium to culture the cells. After the cells were scratched, images were obtained, and the breadths of wound healing were measured at 0 and 24 h.

### Immunohistochemistry

The tumors obtained from mice were fixed with 4% paraformaldehyde for 24 h. After embedding, they were cut into 4-mm-thick sections. Then, we used 10 mmol/L sodium citrate solution to treat the sections. After that, anti-Ki67 antibody (1:100 dilution) was used to incubate the sections at 4°C overnight. Then, we stained the sections utilizing mouse- and rabbit-specific horseradish peroxidase (HRP)/DAB (ABC). Hematoxylin was utilized to counterstain.

### Dual-Luciferase Reporter Gene Assay

Ninety-six-well plates were seeded with cells for 24 h before cotransfection. Each well contains 1 × 10^4^ cells. We used Lipofectamine 3,000 transfection reagent (Thermo Fisher Scientific, Waltham, MA, United States) to cotransfect dual-luciferase reporter vector and miRNA mimics or negative control (NC) mimics into the cells. After incubating the cells for 48 h, we used a dual-luciferase reporter assay system (Promega) according to the protocols of the manufacturer to measure Renilla and firefly luciferase activities.

### RNA Pull-Down

First, we labeled antisense RNA and RNA with biotin. Then, streptavidin beads (Thermo Fisher Scientific, Waltham, MA, United States) were incubated overnight at 4°C. Next, we performed centrifugation at 2,000 rpm for 3 min and used wash buffer to wash them three times. A total of 3 × 10^7^ cells were altogether lysed, and lysates were added to the bead–biotin complex and incubated at 23°C for 1 h. Then, we washed the mixture with wash buffer II and extracted the RNA bound to the beads with TRIzol.

### Western Blot

To perform western blotting, we extracted protein cells with RIPA lysis buffer (Beyotime, Shanghai, China). We added extracts to the gels, and 120 V was used to promote protein migration. Then, the nitrocellulose membranes were transferred with proteins from gels. Tris-buffered saline Tween (TBST) with 5% bovine serum albumin buffer was used to block the membranes. After blocking, we incubated the membrane with primary antibodies (Thermo Fisher Scientific, Waltham, MA, United States) overnight at 4°C. TBST was used to wash the membranes three times, and the membranes were incubated with secondary antibodies at 23°C for 2 h. We detected the protein bands after adding HRP substrate (WBKL0100, Millipore, Guangzhou, China).

### Animal Experiments

After 2 weeks of lentiviral transfection, 1 × 105 U87-luc cells were injected stereotactically into the right basal ganglia of 4–6-week-old female nude mice (*n* = 10, BALB/c-nu, Beijing HFK Bioscience, Beijing, China). We used an *in vivo* imaging system (IVIS Lumina II, United States) to record tumor growth after an intraperitoneal injection of luciferase substrate-D-luciferin (Sinochrome, Shanghai, China).

To establish subcutaneous tumor formation models, we chose 4–6-week-old nude female mice, and each mouse was injected with 2 × 10^7^ transfected cells into the axilla of the right forelimb.

All animal experiments were approved by the Committee of Animal Ethics of Sichuan University.

### Statistical Analysis

We analyzed the data with the GraphPad Prism software. The log-rank test and Kaplan–Meier curves were used to analyze the overall survival. We assessed the statistical significance in two groups using Student’s *t*-test. Pearson correlation test was used to analyze correlations. *p* < 0.05 was considered statistically significant.

## Results

### circRFX3 was Identified as Increased in Glioblastoma

**FIGURE 1:**
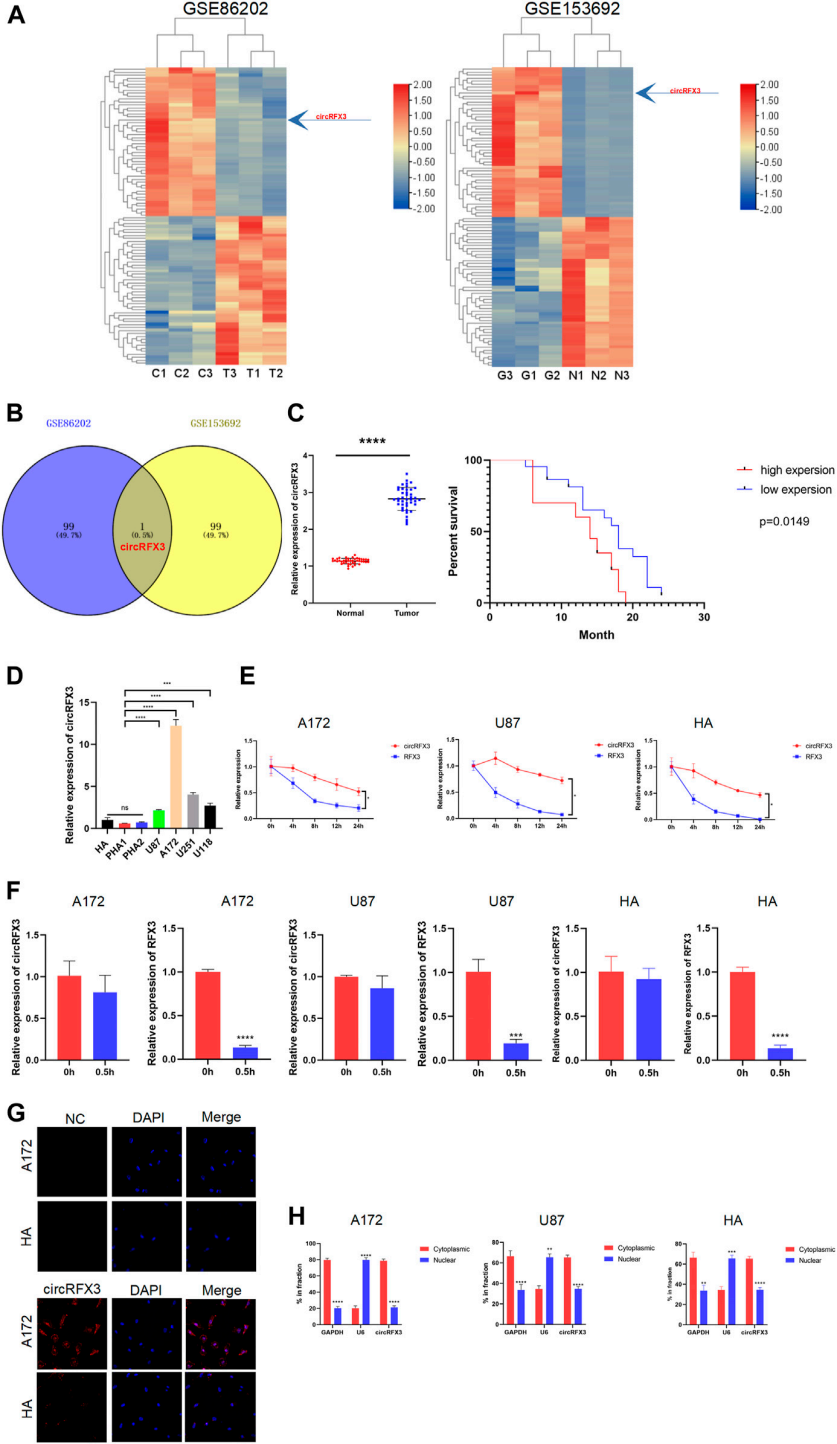
circRFX3 was identified as increased in glioblastoma (GBM) clinical tissues and cell lines. **(A)** The heat maps of the top 50 upregulated and downregulated circRNAs in the GSE86202 and GSE153692 datasets which were selected with bioinformatics technology. **(B)** Venn diagram showing that circRFX3 was the only intersection of the two groups. **(C)** The qPCR results showed that the circRFX3 expression level was much higher in clinical GBM tissues than in paired brain tissues surrounding the tumors. ****p < 0.0001. The expression level of circRFX3 predicted a worse prognosis. **(D)** circRFX3 was remarkably increased in GBM cell lines and human astrocytes. **(E,F)** circRFX3 was more stable than RFX3 mRNA in both the A172 and U87 cell lines. **(G)** The fluorescence *in situ* hybridization results showed that circRFX3 was located mainly in the cytoplasm. **(H)** The qPCR results showed that circRFX3 was located mainly in the cytoplasm of U87 and A172 cells. Statistical analysis was conducted with Student’s *t*-test. *p < 0.05.

To identify specific circRNAs in GBM, we performed circRNA microarray analysis in two GSE datasets (GSE86202 and GSE153692). The top 50 upregulated and downregulated circRNAs in GSE86202 and GSE153692 are listed in Figure 1A. Among these dysregulated circRNAs, circRFX3 (hsa_circRFX3_003, circBank, http://www.circbank.cn) was identified as the only intersection of GSE86202 and GSE153692 (Figure 1B). The expression level of circRFX3 was much higher in GBM tissues from clinical patients than in normal brain tissue around the tumor, and the expression level of circRFX3 predicted a worse prognosis in a group of 42 GBM cases (Figure 1C). The circRFX3 levels were subsequently examined in four GBM cell lines and HA cell line and primary human astrocytes (PHA1 and PHA2) through qRT-PCR. Compared to HAs, PHA1, and PHA2, circRFX3 was remarkably increased in U87, A172, U251, and U118 cells. A172 had the highest circRFX3 expression level, while circRFX3 in U87 cells was expressed at the lowest level (Figure 1D).

Then, we investigated the stabilization and localization of circRFX3 in A172, U87 cells, and HAs. After the GBM cells were treated with actinomycin D at the indicated time points, total RNA from A172 and U87 cells was isolated. Next, the expression levels of circRFX3 and RFX3 mRNA were analyzed by qRT-PCR. By analyzing the outcomes, we concluded that circRFX3 is much more stable in these cells (Figure 1E). Additionally, RNase R could not digest circRFX3 efficiently (Figure 1F). Through fluorescence *in situ* hybridization, we determined that circRFX3 is mainly located in the cytoplasm. This result was further confirmed by nucleus and cytoplasm fraction separation qRT-PCR in A172, U87, cells and HAs (Figures 1G,H). These results suggested that circRFX3 might play an oncogenic role and probably functions as a miRNA sponge, considering its position in the cytoplasm.

### circRFX3 Promoted the Proliferation, Invasion, and Migration of GBM *In Vitro*

**FIGURE 2:**
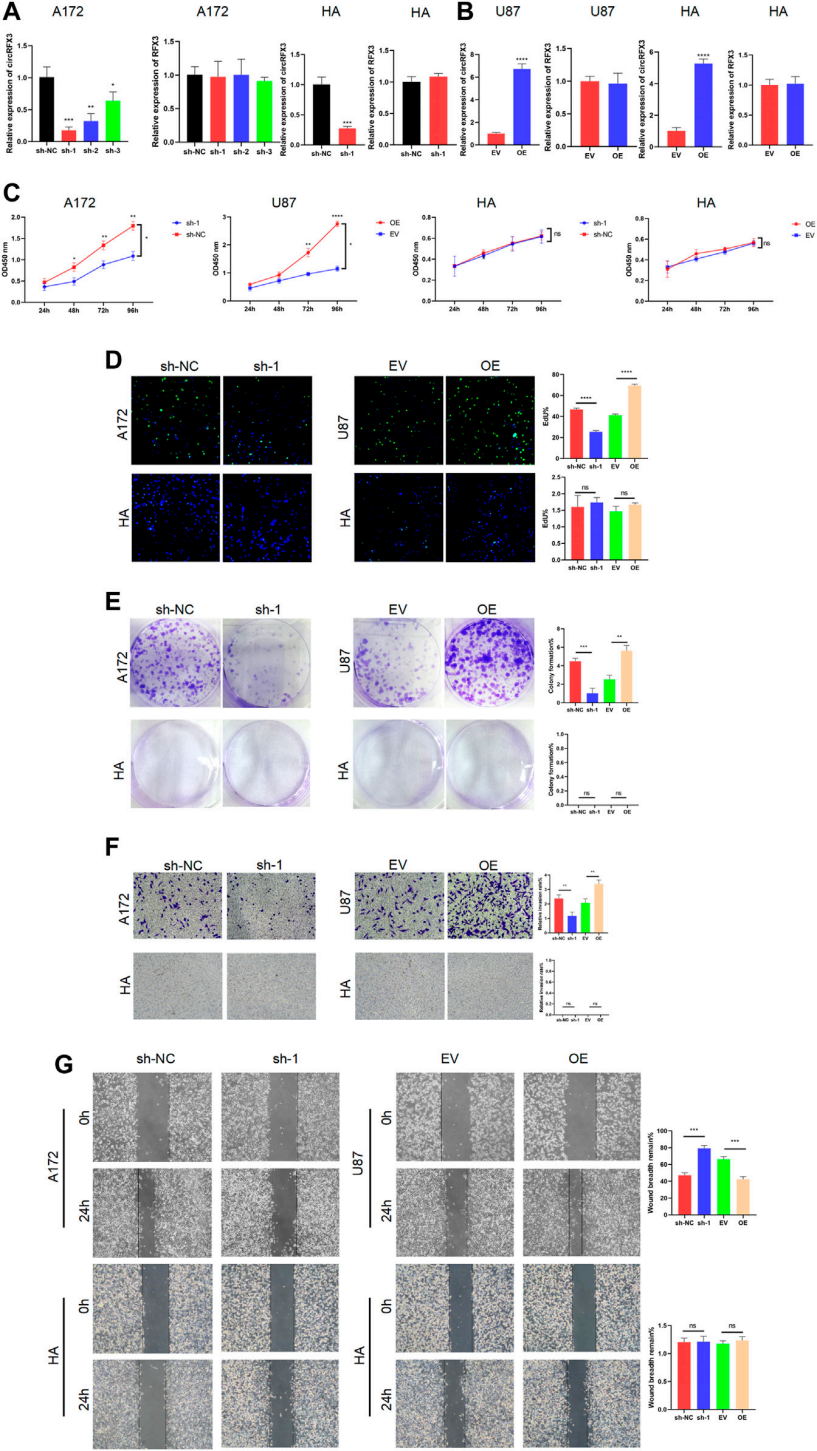
circRFX3 promoted the proliferation, invasion, and migration of glioblastoma *in vitro*. **(A)** circRFX3 expression was obviously inhibited by shRNAs, while RFX3 mRNA did not change. **(B)** circRFX3 expression was obviously promoted, whereas no apparent change in RFX3 mRNA was detected. **(C,D)** The CCK-8 and EdU assays showed that A172 cell proliferation could be suppressed by the inhibition of circRFX3 expression, whereas U87 proliferation could be obviously promoted by the overexpression of circRFX3. Human astrocyte (HA) proliferation could not be influenced by the knockdown or overexpression of circRFX3. **(E)** The colony formation assays showed that the A172 cell colonies were significantly decreased after circRFX3 knockdown, while more U87 cell colonies were formed after circRFX3 overexpression. However, there were no HA colonies formed. **(F,G)** Transwell invasion assays and wound-healing assays showed that suppressing the expression of circRFX3 weakened the invasion and migration abilities of A172 cells, while the invasion and migration of U87 cells were dramatically enhanced after circRFX3 overexpression compared with the HAs. Statistical analysis was conducted with Student’s *t*-test. *p < 0.05.

Given that the circRFX3 expression level was highest in A172 cells but lowest in U87 cells, we chose these two cell lines to knock down or overexpress circRFX3. To investigate the exact effects of circRFX3 in GBM cells, we designed shRNAs targeting the junction sites of circRFX3 and transfected them into A172 cells and HAs. At the same time, we transfected the overexpression vector (OE) and empty vector (EV) of circRFX3 into U87 cells and HAs by lentivirus. The circRFX3 expression was obviously inhibited by shRNAs, while RFX3 mRNA did not change (Figure 2A). Additionally, circRFX3 expression was obviously promoted, whereas no apparent change in RFX3 mRNA was detected (Figure 2B). Among the three shRNAs, the knockdown efficiency of sh-1 was the highest in A172 cells; thus, sh-1 was used in the following experiments. Through analyzing the results of CCK-8 and EdU assays, we concluded that A172 cell proliferation could be suppressed by the inhibition of circRFX3 expression, whereas U87 proliferation could be obviously promoted by overexpression of circRFX3. However, knockdown or overexpression of circRFX3 could not influence the proliferation of HAs (Figures 2C,D). Compared with HAs, A172 cell colonies were significantly decreased after circRFX3 knockdown. In contrast, more U87 cell colonies were formed after circRFX3 overexpression (Figure 2E). Next, we investigated GBM cell invasion and migration. As shown in the results of the invasion and migration assays, suppression of circRFX3 expression weakened the invasion and migration abilities of A172 cells, while the invasion and migration abilities of U87 cells were dramatically enhanced after circRFX3 overexpression compared with HAs (Figures 2F,G).

### circRFX3 Promoted GBM Growth *In Vivo*

**FIGURE 3:**
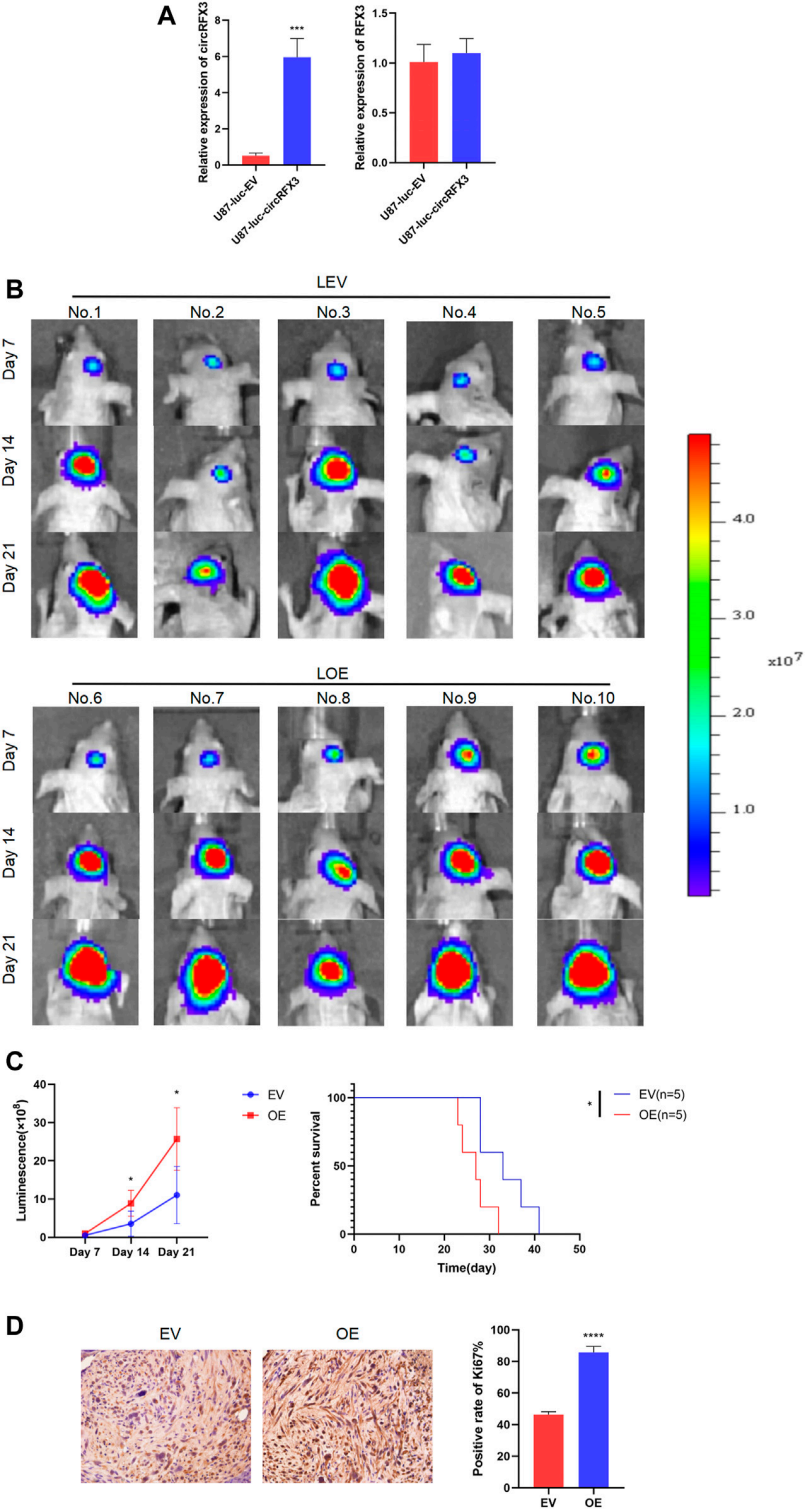
circRFX3 promoted glioblastoma growth *in vivo*. **(A)** The qRT-PCR results showed that circRFX3 was obviously overexpressed in U87-luciferase-circRFX3 overexpression vector (LOE) cells, while RFX3 mRNA did not change. **(B,C)** The outcomes of *in vivo* imaging of small animals showed that tumor growth in the LOE group was clearly accelerated compared with that in the U87-luciferase-empty vector (LEV) group. The survival times of mice in the LOE group were much shorter than those in the LEV group. **(D)** The results of immunohistochemistry staining showed that the expression of Ki67 was obviously boosted by the overexpression of circRFX3. Statistical analysis was conducted with Student’s *t*-test. *p < 0.05.

After investigating the functions of circRFX3 *in vitro*, we decided to determine whether the biological behavior of tumors was regulated by circRFX3 *in vivo*. Hence, we established intracranial xenograft models with nude mice by using stabilized lentivirally transfected U87-luciferase-empty vector (LEV) and U87-luciferase-circRFX3 overexpression vector (LOE) cells. Before the intracranial injection, qRT-PCR was carried out to detect the expression efficacies of circRFX3 in the two transfected cell lines. The results showed that circRFX3 was obviously overexpressed in LOE cells, while RFX3 mRNA did not change (Figure 3A).

Our outcomes indicated that tumor growth in the LOE group (the mice in the LOE group were numbered 6, 7, 8, 9, and 10) was clearly accelerated compared with that in the LEV group (the mice in the LEV group were numbered 1, 2, 3, 4, and 5). In addition, we demonstrated that the survival times of mice in the LOE group were much shorter than those in the LEV group (Figures 3B,C). Furthermore, the expression of Ki67 was obviously boosted by circRFX3 overexpression (Figure 3D). These outcomes indicated that circRFX3 boosted tumor growth *in vivo*.

### circRFX3 Acted as miR-587 Sponge in GBM Cells

**FIGURE 4:**
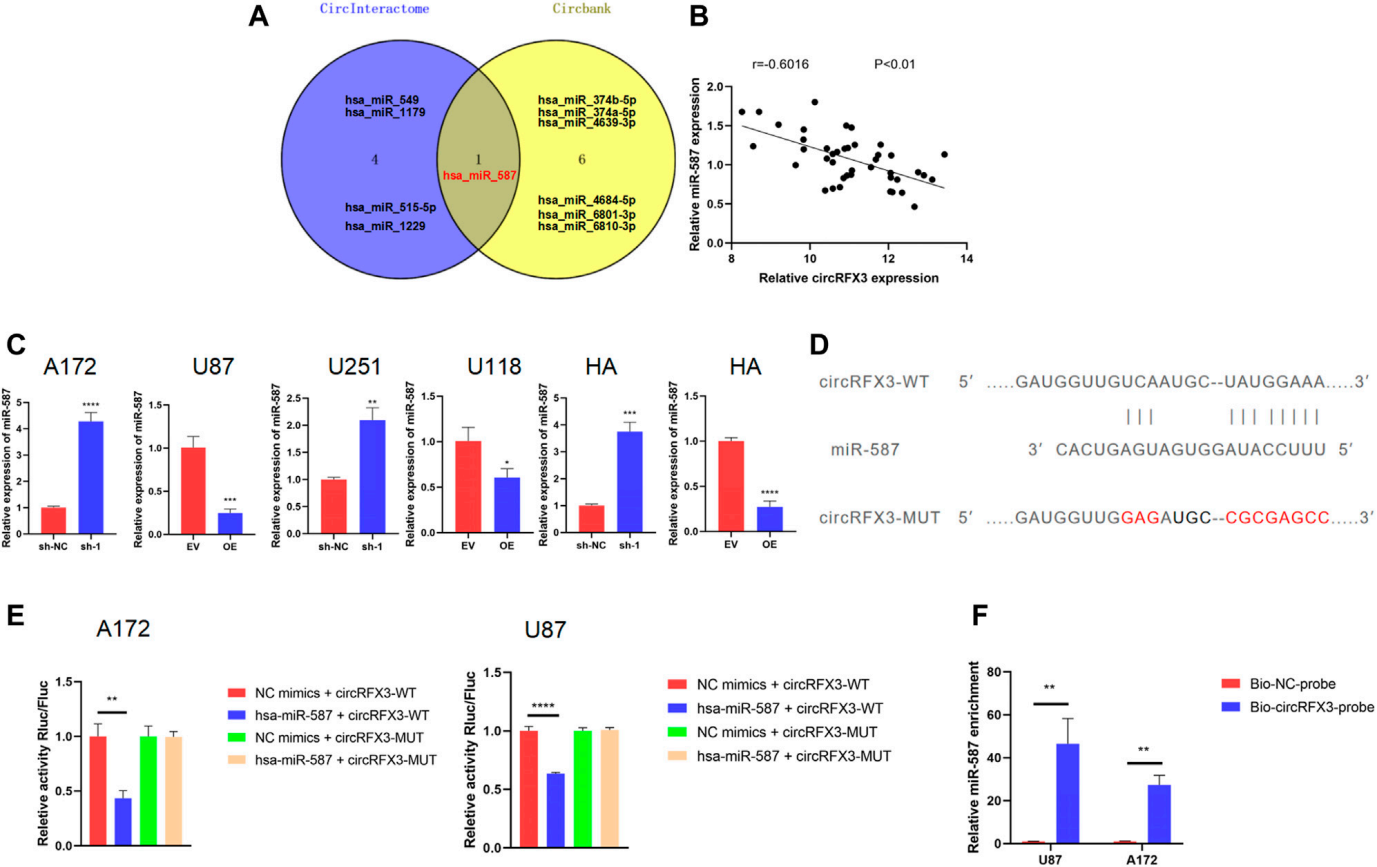
circRFX3 could sponge miR-587 in glioblastoma (GBM) cells. **(A)** Venn diagram showing that hsa_miR_587 was the only intersection of the two databases. **(B)** circRFX3 expression was inversely associated with miR-587 in clinical GBM samples. **(C)** miR-587 was significantly upregulated after circRFX3 knockdown in A172 and U251 cells and human astrocytes (HAs), whereas miR-587 was downregulated due to circRFX3 overexpression in U87 and U118 cells and HAs. **(D)** The wild-type (WT) and mutated-type binding sites for miR-587 in circRFX3. **(E)** The results of the dual-luciferase reporter gene assays showed that the relative luciferase activity of the cells containing the WT miR-587 binding sites in circRFX3 could be obviously inhibited by transfecting miR-587 mimics. The relative luciferase activity of the cells that contained the mutated binding site did not change. **(F)** After the RNA pull-down assays, the qRT-PCR results showed that endogenous miR-587 was a significantly enriched circRFX3 probe. Statistical analysis was conducted with Student’s *t*-test. *p < 0.05.

Following these tests, we sought to determine the approach through which the proliferation, invasion, and migration of GBM were promoted by circRFX3. A number of studies have confirmed that circRNAs can function as miRNA sponges. (Wang et al., 2021a; Shi et al., 2021; Zheng et al., 2021). Given that circRFX3 exists mainly in the cytoplasm, we speculated that circRFX3 might influence GBM biological behavior by sponging miRNAs. We used circInteractome (https://circinteractome.irp.nia.nih.gov/) and circBank (http://www.circbank.cn/) to identify which miRNAs could be sponged by circRFX3. Surprisingly, only one miRNA (hsa_miR_587) was predicted by the two databases (Figure 4A). Additionally, circRFX3 expression was inversely associated with miR-587 in clinical GBM samples (Figure 4B). Furthermore, miR-587 was significantly upregulated after circRFX3 knockdown in A172 and U251 cells, whereas miR-587 was downregulated due to circRFX3 overexpression in U87 and U118 cells (Figure 4C).

To verify the database predictions, we inserted the psiCHECK2 3′UTR plasmid with the sequence of circRFX3 (wild type, WT) to establish a dual-luciferase reporter system. After that, a mutated sequence was cloned into the psiCHECK2 plasmid (MUT) 3′UTR, whose binding sites for miR-587 in circRFX3 were mutated (Figure 4D). Next, to confirm whether miR-587 could directly bind to circRFX3, we performed dual-luciferase reporter assays in both A172 and U87 cells. As Figure 4E shows, the relative luciferase activity of the cells containing the WT miR-587 binding sites in circRFX3 could be obviously inhibited by the transfection of miR-587 mimics. However, the relative luciferase activity of the vector containing the mutated binding site did not change. To further validate the binding of miR-587 to circRFX3, we also performed RNA pull-down assays in A172 and U87 cells. The qRT-PCR results showed that endogenous miR-587 was a significantly enriched circRFX3 probe (Figure 4F).

### circRFX3 Enhanced the Malignant Behavior of GBM Cells by Suppressing miR-587 *In Vitro* and *In Vivo*

**FIGURE 5:**
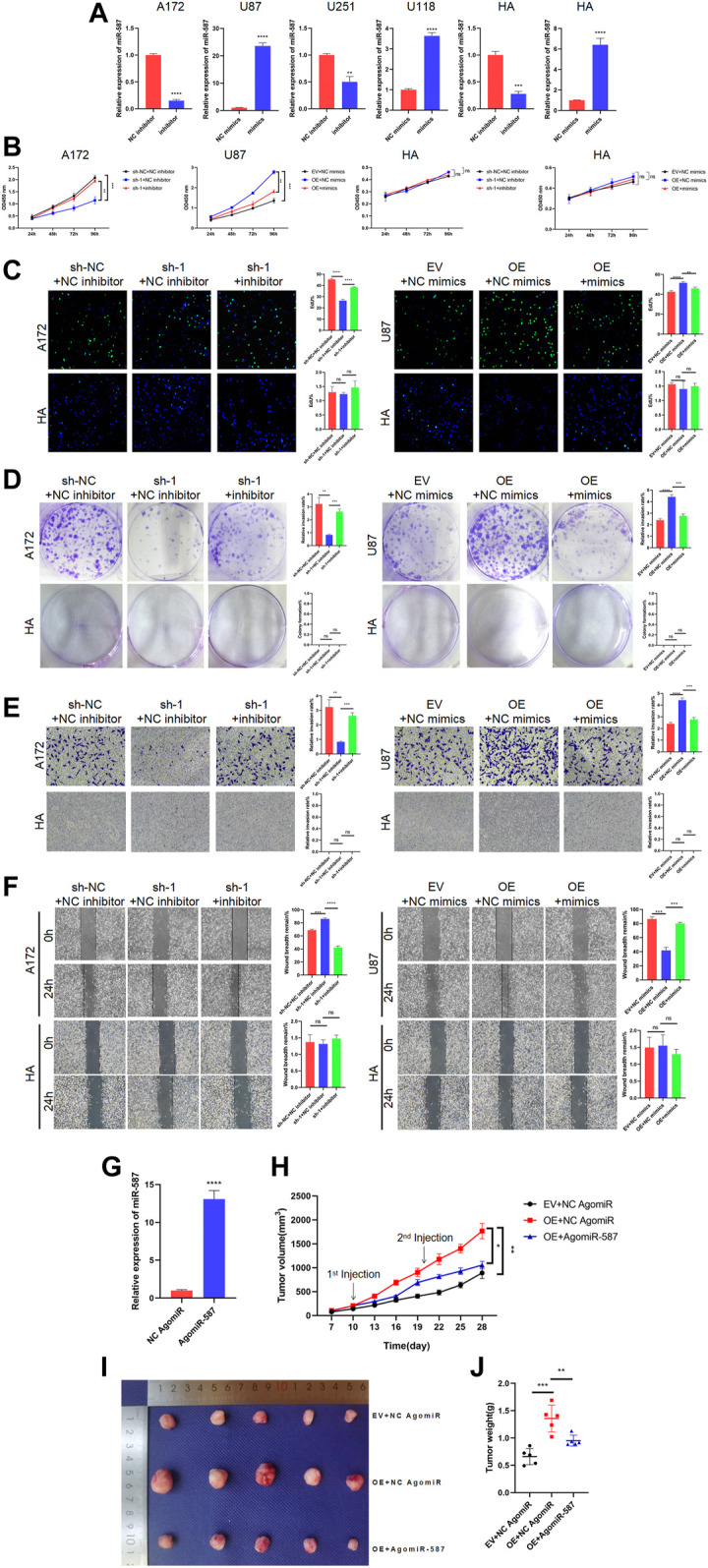
circRFX3 enhanced the malignant behavior of glioblastoma (GBM) cells by suppressing miR-587 *in vitro* and *in vivo*. **(A)** miR-587 mimics increased the miR-587 expression, while miR-587 inhibitor decreased the miR-587 expression in GBM cells and human astrocytes (HAs). **(B–D)** CCK-8, EdU, and colony formation experiments demonstrated that the circRFX3-sponging effect on miR-587 partially rescued the proliferation inhibition caused by circRFX3 depletion in A172 cells, whereas the promotion of cell proliferation led by circRFX3 overexpression in U87 cells was reversed by increasing miR-587. However, the knockdown or overexpression of miR-587 had no effect on HAs. **(E,F)** The results of transwell invasion and wound-healing assays showed that the miR-587 inhibitor partially attenuated the reduced invasion and migration induced *via* circRFX3 knockdown, while miR-587 mimics had the opposite effect. However, the knockdown or overexpression of miR-587 had no effect on HAs. **(G)** AgomiR-587 could increase the miR-587 expression. **(H)** The growth curve showed that the tumors injected with AgomiR-587 had a slower growth rate than the negative control (NC) group. **(I,J)** The tumors injected with AgomiR-587 had smaller sizes and weights than those in the NC AgomiR group. Statistical analysis was conducted with Student’s *t*-test. *p < 0.05.

We next performed a series of rescue assays to investigate whether circRFX3 boosts the malignant biological behavior of GBM by sponging miR-587. First, we designed miR-587 mimics and miR-587 inhibitors. As shown in Figure 5A, miR-587 mimics increased miR-587 expression, while miR-587 inhibitors decreased miR-587 expression in GBM cells and HAs. CCK-8, EdU, and colony formation experiments demonstrated that the circRFX3-sponging effect on miR-587 partially rescued the proliferation inhibition caused by circRFX3 depletion in A172 cells, whereas the promotion of cell proliferation led by circRFX3 overexpression in U87 cells was reversed by increasing miR-587. However, knockdown or overexpression of circRFX3 could not influence the proliferation of HAs (Figures 5B–D). In addition, the miR-587 inhibitor partially attenuated the reduced invasion and migration induced *via* circRFX3 knockdown, while miR-587 mimics had the opposite effect, as indicated by the invasion and migration assays (Figures 5E,F).

To explore the effects of miR-587 *in vivo*, we designed AgomiR-587, which increased the efficient expression of miR-587 (Figure 5G). Then, nude mice were subcutaneously injected with stabilized U87 cells transfected with EV or OE. As demonstrated in Figure 5H, after tumor formation, we separately injected the tumors with NC AgomiR and AgomiR-587 every 10 days, and tumor sizes were measured every 3 days from day 7 to 28. A slower growth rate was represented in the group of tumors injected with AgomiR-587 compared with the NC group (Figure 5I). The tumors were harvested and photographed on day 28, and the tumors injected with AgomiR-587 had smaller sizes and weights than those in the NC group (Figure 5J). These data clearly demonstrated that circRFX3 enhanced the malignant behavior of GBM cells by suppressing miR-587.

### PDIA3 Was a Downstream Target of miR-587

**FIGURE 6:**
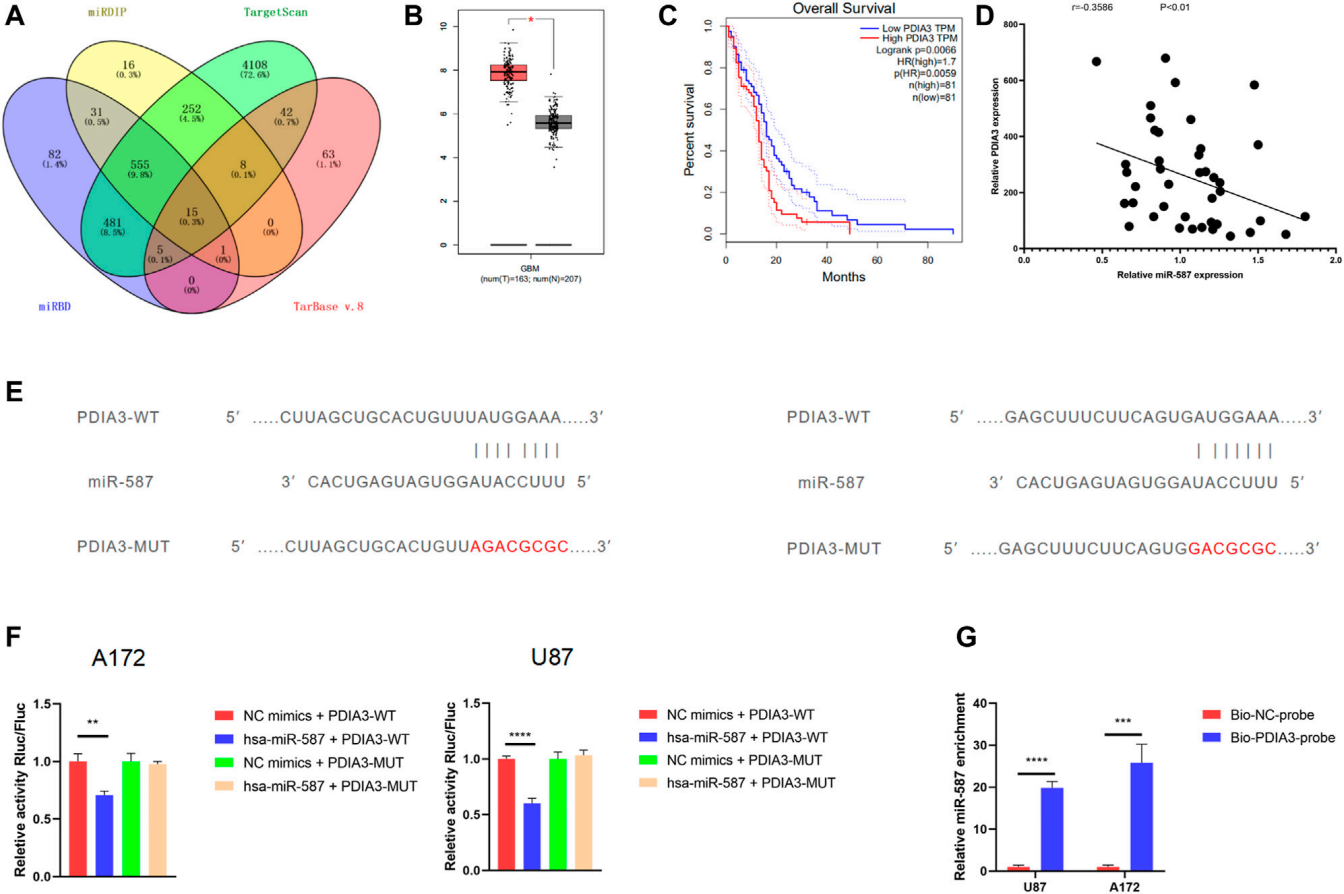
PDIA3 was a downstream target of miR-587. **(A)** The Venn diagram shows 15 potential targets of miR-587 by overlapping the outcomes of the four algorithms. **(B)** The analysis of The Cancer Genome Atlas glioblastoma (GBM) datasets showed that PDIA3 had an obviously higher expression level in 163 GBM clinical tissues than in 207 normal brain tissues. **(C)** Kaplan–Meier survival analysis showed that PDIA3 expression was obviously correlated with worse overall survival. **(D)** The qRT-PCR results showed that PDIA3 expression was negatively correlated with miR-587 in GBM clinical samples. **(E)** The wild-type (WT) and mutated-type binding sites for miR-587 in PDIA3 mRNA. **(F)** The results of dual-luciferase reporter gene assays showed that the relative activity of luciferase in U87 and A172 cells transfected with the WT 3′UTR sequence was obviously suppressed by miR-587 mimics. However, the relative luciferase activity did not change after the transfection of miR-587 mimics in U87 and A172 cells with the MUT 3′UTR sequence. **(G)** After the RNA pull-down assay, qPCR showed that the 3′UTR of PDIA3 mRNA was significantly enriched by miR-587. Statistical analysis was conducted with Student’s *t*-test. *p < 0.05.

To determine whether circRFX3 boosts the expression of downstream targets by sponging miR-587, we sought out 15 potential targets (SLC7A11, NAB1, REEP5, NFAT5, CAPRIN1, RNF169, PDIA3, DST, MBNL1, NAMPT, FGF5, DICER1, SEC23A, SSR1, and POFUT1) of miR-587 by overlapping the outcomes of predictions by four algorithms (miRDIP, http://ophid.utoronto.ca/mirDIP/); miRBD, http://mirdb.org/; Tarbase v.8 http://carolina.imis.athena-innovation.gr/diana_tools/web/; and TargetScan, http://www.targetscan.org/) (Figure 6A). Then, the GBM dataset from the TCGA (https://www.cancer.gov/about-nci/organization/ccg/research/structural-genomics/tcga) database was analyzed, and we observed that PDIA3 had an obviously higher expression level in 163 GBM clinical tissues than in 207 normal brain tissues (Figure 6B). In addition, we used TCGA GBM datasets to conduct Kaplan–Meier survival analysis and found that PDIA3 expression was obviously correlated with worse overall survival (Figure 6C). However, compared with normal brain tissues, the outcomes of Kaplan–Meier overall survival analysis suggested that the expression of the other 14 genes in GBM tissues was not significantly associated with overall patient survival. Furthermore, the qRT-PCR results showed that PDIA3 expression was negatively correlated with miR-587 in GBM clinical samples (Figure 6D). Based on these data, we speculated that PDIA3 is a downstream target of miR-587.

A dual-luciferase reporter assay was performed to clarify whether the PDIA3 mRNA 3′UTR is a binding site of miR-587 in GBM cells. There were two predicted binding sites on PDIA3 (Figure 6E). We inserted the psiCHECK2 plasmid 3′UTR (WT) with the sequence of PDIA3. After that, a mutated sequence was cloned into the psiCHECK2 plasmid (MUT) 3′UTR. The relative luciferase activity in U87 and A172 cells transfected with the WT 3′UTR sequence (WT) was obviously suppressed by miR-587 mimics. However, the relative luciferase activity did not change after the transfection of miR-587 mimics in U87 and A172 cells with the MUT 3′UTR sequence (Figure 6F). Furthermore, an RNA pull-down assay was performed. We found that the 3′UTR of PDIA3 mRNA was significantly enriched by miR-587 (Figure 6G). These outcomes indicated that the 3′UTR sequence of PDIA3 could be bound by miR-587.

### PDIA3 Played a Tumor-Promoting Role in GBM Cells, and circRFX3 Activated the Wnt/β-Catenin Pathway Through the miR-587/PDIA3 Axis

**FIGURE 7:**
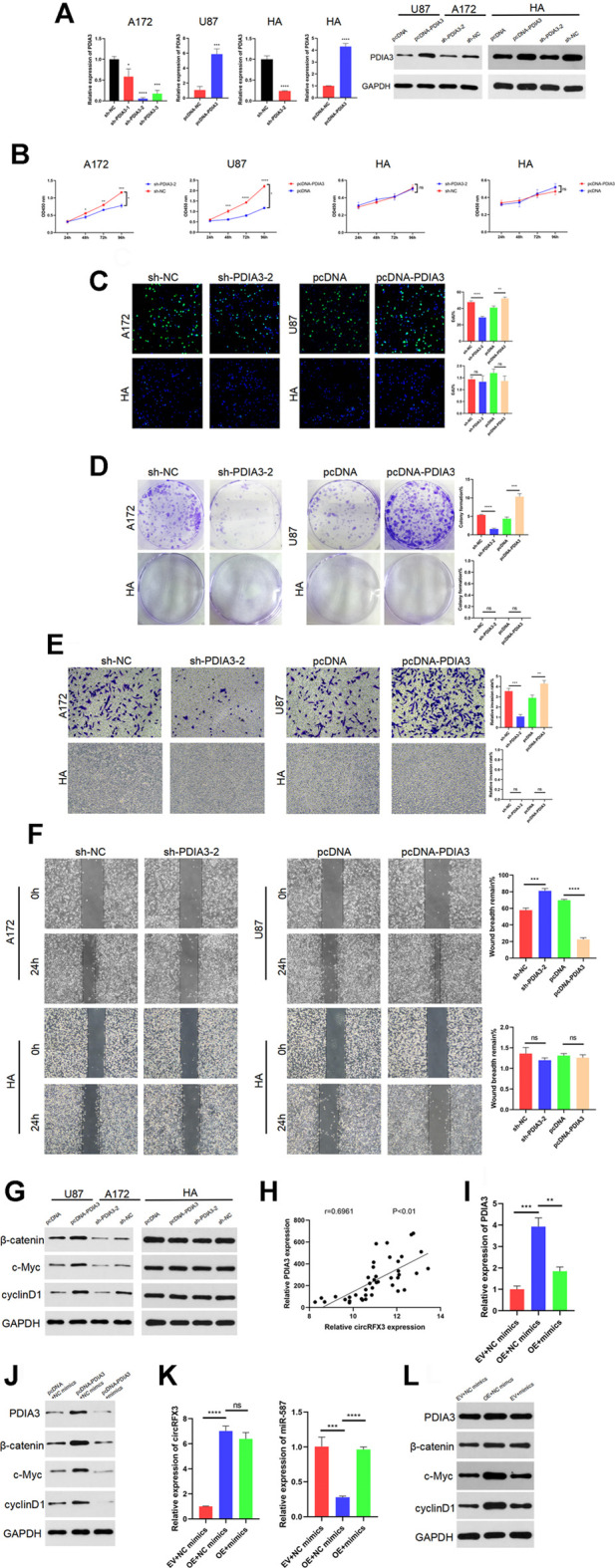
PDIA3 played tumor-promoting roles in glioblastoma (GBM) cells, and circRFX3 regulated the miR-587/PDIA3/β-catenin axis. **(A)** As the qPCR and western blotting results showed, PDIA3 expression was clearly suppressed by sh-PDIA3-1, sh-PDIA3-2, and sh-PDIA3-3 in A172 cells, and PDIA3 was obviously overexpressed in U87 cells and human astrocytes (HAs). **(B–D)** In EdU, CCK-8, and colony-formation assays, PDIA3 knockdown weakened the viability of A172 cells, while PDIA3 upregulation significantly promoted cell viability in U87 cells. However, the viability of HAs was not influenced by the knockdown or overexpression of PDIA3. **(E,F)** Transwell invasion and wound-healing assays revealed that PDIA3 knockdown inhibited GBM cell invasion and migration, but PDIA3 overexpression had the opposite effect. **(G)** Western blotting demonstrated that the Wnt/β-catenin pathway was suppressed after PDIA3 knockdown in A172 cells, whereas the pathway was activated by PDIA3 overexpression in U87 cells. However, the Wnt/β-catenin pathway was not influenced by the knockdown or overexpression of PDIA3 in HAs. **(H)** The qPCR results showed a positive correlation between PDIA3 and circRFX3 in human GBM samples. **(I,J)** As shown by the western blotting and qRT-PCR results, miR-587 partially reversed the cancer-promoting functions of PDIA3. **(K,L)** miR-587 partially reversed the promoting functions of circRFX3 on PDIA3 and the Wnt/β-catenin pathway in GBM cells. Statistical analysis was conducted with Student’s *t*-test. *p < 0.05.

To determine the effects of PDIA3 in GBM, three short hairpin RNAs (sh-PDIA3-1, sh-PDIA3-2, and sh-PDIA3-3) were designed and transfected into A172 cells and HAs. We also designed PDIA3 overexpression plasmids (pcDNA-PDIA3) and empty plasmids (pcDNA) and transfected them into U87 cells and HAs. As Figure 7A shows, PDIA3 expression was apparently suppressed by sh-PDIA3-1, sh-PDIA3-2, and sh-PDIA3-3 in A172 cells, and PDIA3 was obviously overexpressed in U87 cells and HAs. sh-PDIA3-2 was used for subsequent experiments. In EdU, CCK-8, and colony formation assays, PDIA3 knockdown weakened the viability of A172 cells, while PDIA3 upregulation significantly promoted cell viability in U87 cells compared with HAs (Figures 7B–D). Furthermore, invasion and migration assays revealed that PDIA3 knockdown inhibited GBM cell invasion and migration, but PDIA3 overexpression had the opposite effect (Figures 7E,F). Based on these outcomes, PDIA3 clearly boosted GBM cell proliferation, invasion, and migration.

Furthermore, western blotting demonstrated that the Wnt/β-catenin pathway was suppressed after PDIA3 knockdown in A172 cells, whereas the pathway was activated by PDIA3 overexpression in U87 cells. However, the Wnt/β-catenin pathway was not influenced by knockdown or overexpression of PDIA3 in HAs (Figure 7G). In addition, we confirmed the negative correlation between miR-587 and circRFX3/PDIA3 (Figures 5B, 6D) and the positive correlation between PDIA3 and circRFX3 in human GBM samples (Figure 7H). Taken together, these results led us to speculate that circRFX3 promoted GBM cell viability, invasion, and migration by sponging miR-587 and upregulating PDIA3 expression to regulate the Wnt/β-catenin pathway. To confirm this speculation, we designed rescue experiments that used miR-587 mimics. As shown in the qRT-PCR and western blotting results (Figures 7I,J), miR-587 partially reversed the cancer-promoting functions of PDIA3. Figures 7K,L showed that miR-587 partially reversed the promoting functions of circRFX3 on PDIA3 and the Wnt/β-catenin pathway in GBM cells.

## Discussion

In this study, we investigated the role of a novel circRNA (circRFX3) in the progression of GBM. Our outcomes demonstrated that the circRFX3 expression levels were increased in GBM and indicated the worse survival of patients with GBM. Additionally, circRFX3 sponges miR-587 and upregulates the PDIA3/β-catenin axis.

Circular RNAs influence the oncogenesis and progression of numerous tumors, including GBM—for instance, circ_0005774 promotes the proliferation of acute myeloid leukemia cells. (Li et al., 2021). Furthermore, the inhibition of circ_0000326 weakens cervical cancer cell migration, invasion, and proliferation (Wang et al., 2021b). circ_0002162 promotes the malignant development of tongue cancer (Zhang et al., 2021a). Additionally, the overexpression of circ_0005927 restrains the development of colorectal carcinoma (Yu et al., 2021b). Moreover, circ_0015326 facilitates the invasion, migration, and proliferation of ovarian cancer (Zhang et al., 2021b). With the help of bioinformatics, we speculated that circRFX3 played an essential role in GBM. As expected, circRFX3 was confirmed to be overexpressed in GBM cells and clinical tumor samples, and the results of various *in vitro* and *in vivo* assays suggested that circRFX3 contributes to proliferation, invasion, and migration, indicating that circRFX3 acts as a therapeutic target and a prognostic factor in GBM.

A number of studies have demonstrated that microRNAs inhibit the progression of multiple tumors—for instance, miR-587 inhibits the proliferation of prostate carcinoma cells by targeting downstream protein (Du and Gao, 2021). Similarly, miR-587 plays a tumor-inhibiting role in hepatocellular cancer (Chen et al., 2020). The outcomes of the present study showed that miR-587 expression was inversely associated with circRFX3 expression in GBM cells and clinical tumor tissues. An increasing number of studies have confirmed that circular RNAs act as competitive endogenous RNAs (ceRNAs) of miRNAs—for instance, circPARP4 facilitates glioblastoma development by sponging miR-587 (Zhou et al., 2021). Additionally, circ_102049 promotes pancreatic ductal adenocarcinoma development by sponging miR-455-3p21 (Zhu et al., 2021). In the present study, two potential binding sites between circRFX3 and miR-587 were identified by using bioinformatics technology, and we hypothesized that miR-587 may be a downstream target of circRFX3 in GBM. Then, dual-luciferase reporter assays were performed to confirm the binding sites between the two RNAs. Our results showed that circRFX3 weakened the inhibitory functions of miR-587. Hence, circRFX3 exercised its biological functions by sponging miR-587.

PDIA3 was proven to participate in regulating proliferation, invasion, and migration in many tumors—for instance, PDIA3 in GBM regulates macrophage/microglia pro-tumor activation (Chiavari et al., 2020). Additionally, PDIA3 promotes the invasion and proliferation of acute myeloid leukemia (Ye et al., 2018) and suppresses the migration and proliferation of keratinocytes—for example, PDIA3 could promote the growth of hepatocellular cancer. Moreover, PDIA3 upregulates cell proliferation in cutaneous malignant melanoma. (Su et al., 2016). In the present study, we verified through *in vitro* and *in vivo* assays that PDIA3 boosted cell proliferation, invasion, and migration. Additionally, PDIA3 activates the Wnt/β-catenin pathway in GBM. With the help of bioinformatics technology, the binding sites between miR-587 and PDIA3 were predicted, which led us to further investigate whether circRFX3 indirectly upregulated PDIA3 by sponging miR-587 and activating the Wnt/β-catenin pathway. The final outcomes of our experiments are consistent with our speculation.

**FIGURE 8:**
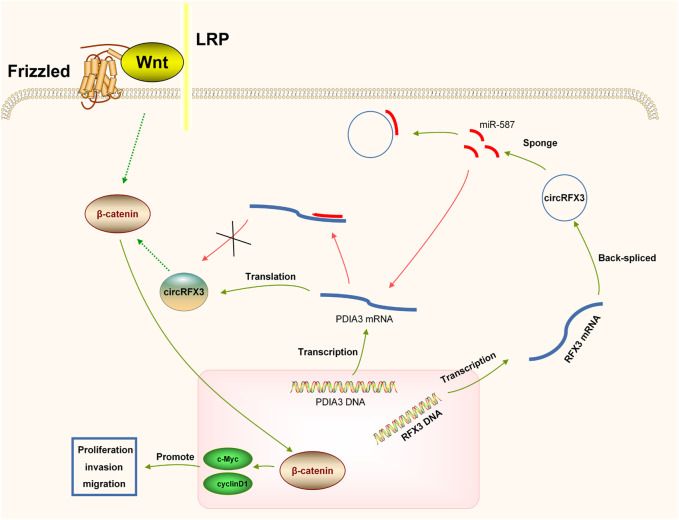
circRFX3 could act as a cancer-promoting circRNA to boost the development of glioblastoma and regulate the miR-587/PDIA3/β-catenin axis.

In summary, by regulating the miR-587/PDIA3/β-catenin axis, circRFX3 enhances proliferation, invasion, and migration (Figure 8). These results might provide a better understanding of molecular therapy. However, the number of clinical samples in this study might be insufficient. Thus, it is essential to further investigate the functions of genes in additional clinical trials. Furthermore, more *in vivo* assays are required to further study the circRFX3/miR-587/PDIA3/β-catenin axis in GBM development.

## Data Availability

The datasets presented in this study can be found in online repositories. The names of the repository/repositories and accession number(s) can be found below: https://www.ncbi.nlm.nih.gov/geo/, GSE86202; https://www.ncbi.nlm.nih.gov/geo/, GSE153692.
